# Ethanol-Induced White Matter Atrophy Is Associated with Impaired Expression of Aspartyl-Asparaginyl-*β*-Hydroxylase (ASPH) and Notch Signaling in an Experimental Rat Model

**DOI:** 10.4303/jdar/236033

**Published:** 2017-08-23

**Authors:** Ming Tong, Howard Gonzalez-Navarrete, Tyler Kirchberg, Billy Gotama, Emine B. Yalcin, Jared Kay, Suzanne M. de la Monte

**Affiliations:** 1 Liver Research Center, Division of Gastroenterology and Department of Medicine, Rhode Island Hospital, Warren Alpert Medical School of Brown University, Providence, RI 02903, USA; 2 Departments of Neurology, Neurosurgery, and Pathology, Rhode Island Hospital, Warren Alpert Medical School of Brown University, Providence, RI 02903, USA; 3 Rhode Island Hospital, Warren Alpert Medical School of Brown University, Providence, RI 02903, USA; 4 Molecular Pharmacology and Biotechnology Graduate Program, Brown University, Providence, RI 02912, USA; 5 Brown University, Providence, RI 02912, USA

**Keywords:** alcohol, neurodegeneration, oligodendrocytes, aspartyl-asparaginyl-*β*-hydroxylase, white matter, Notch, hypoxia-inducing factor 1-alpha

## Abstract

Alcohol-induced white matter (WM) degeneration is linked to cognitive-motor deficits and impairs insulin/insulin-like growth factor (IGF) and Notch networks regulating oligodendrocyte function. Ethanol downregulates Aspartyl-Asparaginyl-*β*-Hydroxylase (ASPH) which drives Notch. These experiments determined if alcohol-related WM degeneration was linked to inhibition of ASPH and Notch. Adult Long Evans rats were fed for 3, 6 or 8 weeks with liquid diets containing 26% ethanol (caloric) and in the last two weeks prior to each endpoint they were binged with 2 g/kg ethanol, 3*×*/week. Controls were studied in parallel. Histological sections of the frontal lobe and cerebellar vermis were used for image analysis. Frontal WM proteins were used for Western blotting and duplex ELISAs. The ethanol exposures caused progressive reductions in frontal and cerebellar WM. Ethanol-mediated frontal WM atrophy was associated with reduced expression of ASPH, Jagged 1, HES-1, and HIF-1*α*. These findings link ethanol-induced WM atrophy to inhibition of ASPH expression and signaling through Notch networks, including HIF-1*α*.

## 1. Introduction

Consequences of alcohol abuse and addiction are among the costliest healthcare problems in the world. In the United States, alcohol abuse is the third leading preventable cause of death (88,000/year) [1,2,3]. Furthermore, alcohol-related brain disease (ARBD) characterized by neurobehavioral abnormalities and cognitive deficits including impairments in executive function [4] can disrupt family and social relationships and progress to dementia and disability [5,6]. A consistent neuroanatomical substrate of ARBD including cognitive impairment is brain atrophy [6,7,8,9,10] with selective degeneration of white matter (WM) [11,12,13,14] due in part to myelin loss [8,11,15,16,17].

The severity of ARBD-associated WM atrophy correlates with maximum daily and lifetime alcohol exposures [8,13,18]. Although the WM atrophy can be diffuse, ARBD most prominently targets the corpus callosum and prefrontal, temporal, and cerebellar WM [8,19], corresponding with the sustained adverse effects on executive, cognitive, and motor functions. Ultrastructural studies of an experimental model demonstrated that WM atrophy following chronic heavy alcohol exposure is mediated by combined effects of demyelination, dysmyelination, and axonal degeneration [20]. The finding that chronic ethanol exposures broadly alter oligodendrocyte myelin-associated gene expression [21,22] suggests that the neurotoxic and degenerative effects of alcohol include oligodendrocyte dysfunction.

Oligodendrocytes generate and maintain central nervous system (CNS) myelin via differential expression and activation of enzymes needed for its biosynthesis, turnover, and degradation [23,24,25,26]. Oligodendrocyte survival and function, including myelin homeostasis, are supported by insulin and insulin-like growth factor, type 1 (IGF-1) signaling [27,28,29,30]. For example, in vivo overexpression of IGF-1 increases brain size, oligodendrocyte abundance, and myelin content [31,32], whereas depletion of IGF-1 or its receptor genes, or overexpression of IGF binding proteins, impairs brain growth and oligodendrocyte myelin maintenance and maturation [33,34]. One of the main adverse effects of chronic and/or binge ethanol exposures is to inhibit brain insulin and IGF-1 signaling through pathways that regulate cell survival, gene and protein expression, growth, metabolism, and plasticity [35, 36,37,38,39,40,41,42,43,44,45]. Sustained inhibition of insulin/IGF-1 signaling alters the expression of downstream target genes and proteins that regulate important cellular functions. Aspartyl-asparaginyl-*β*-hydroxylase (ASPH; formally abbreviated AAH) is one such insulin/IGF-1 regulated gene that is inhibited by ethanol [36,46,47,48].

ASPH is an *~* 86 kD transmembrane phosphoprotein that is expressed on cell surface and endoplasmic reticulum membranes [49,50,51]. ASPH is physiologically cleaved into *~* 30–34 kD N-terminal and *~* 52–54 kD C-terminal fragments. ASPH protein physically interacts with Notch and Jagged [52,53] and its C-terminal catalytic domain hydroxylates *β*-carbons of Asp and Asn residues in their EGF-like domains [49,51]. Attendant activation of Notch pathways promotes cell motility and adhesion of immature and neoplastic cells [49,52,54,55]. In addition, ASPH signals through hypoxia-inducible factor 1 alpha (HIF-1*α*), driving cell motility under conditions of oxidative stress [54, 56,57].

ASPH’s roles in cell motility, adhesion, and tissue invasion have been largely characterized in malignant neoplastic cells, immature brains, and placenta. In contrast, little is known about its potential functions in mature tissues and postmitotic cells in which ASPH is generally expressed at low levels. However, relevant clues may be derived from a previous human study of cerebral autosomal dominant arteriopathy with subcortical infarcts and leukoencephalopathy (CADASIL) [58]. CADASIL is a predominantly cerebral WM degenerative disease in which the Notch 3 gene on Chromosome 19 is mutated and progressive loss of myelinated fibers culminates in dementia [59]. In CADASIL, WM ASPH expression is significantly reduced along with profound suppression of mRNAs encoding Notch, insulin, IGF-1 and IGF-2 receptors, mature oligodendrocyte myelin-associated genes, and oligodendrocyte transcription factors [58]. These observations suggest that the targeting of WM oligodendrocytes in CADASIL is linked to impaired expression of Notch and ASPH, and reduced signaling through insulin/IGF pathways. However, alcohol-induced WM atrophy is also associated with inhibition of insulin/IGF signaling and altered expression of oligodendrocyte myelin-associated genes and transcription factors. This study explores the potential role of impaired ASPH and Notch signaling as mediators of progressive ARBD-associated WM atrophy.

## 2. Methods

### 2.1. Experimental model

Adult 6-week-old male Long Evans rats were fed with isocaloric liquid diets containing 0% or 26% (caloric) for 3, 6 or 8 weeks (*n* = 6–8/group). To generate a chronic+binge ethanol model, during the last two weeks of liquid diet feeding, the ethanol group was binged with 2 g/kg ethanol by intraperitoneal (IP) injection on Mondays, Wednesdays, and Fridays; controls were treated with IP saline. Previous studies showed that the chronic+binge model effectively produces WM degeneration and cognitive impairment in rats [22,44]. Rats were sacrificed by isoflurane inhalation. Freshly harvested brains were systematically microdissected to obtain pretemporal coronal slices of the frontal lobes and midsagittal sections of cerebella. One frontal lobe and one hemicerebellum from each rat was frozen on dry ice and stored at −80 °C, and corresponding samples from the opposite hemispheres were immersion fixed in 10% neutral buffered formalin for paraffin embedding. Rats were housed under standardized conditions with 12 h light/dark cycles, and controlled temperature (70 °F–74 °F). These experiments were approved by the Institutional Animal Care and Use Committee (IACUC) at the Lifespan/Rhode Island Hospital and the protocols followed guidelines established by the National Institutes of Health.

### 2.2. Histological and image analysis studies

Formalin-fixed frontal lobes and cerebella were embedded in paraffin. Histological sections (4 *μ*m thick) generated 5 mm from the temporal pole for the frontal lobe and 5 mm lateral to the midline vermis of the cerebellum were stained with Luxol fast blue, hematoxylin, and eosin (LHE) and used for image analysis. ImageJ/Fiji software (NIH) was used to measure cross-sectional areas of frontal and cerebellar cortex and WM, and assess cellularity within the granule and Purkinje cell layers of the cerebellum. All slide analyses were performed under code.

### 2.3. Preparation of protein homogenates

The protein expression studies were limited to the frontal lobe because it was not feasible to microdissect cerebellar WM. Frontal lobe WM was homogenized in buffer containing 50 mM Tris (pH 7.5), 150 mM NaCl, 5 mM EDTA (pH 8.0), 50 mM NaF, 0.1% Triton X-100, and protease (1 mM PMSF, 0.1 mM TPCK, 1 mg/mL aprotinin, 1 mg/mL pepstatin A, 0.5 mg/mL leupeptin, 1 mM NaF, 1 mM Na_4_P_2_O_7_) and phosphatase (2 mM Na_3_VO_4_) inhibitors using a TissueLyser II (Qiagen, Germantown, MD, USA) with 5 mm stainless steel beads as described. Supernatants obtained after centrifuging the samples at 14,000 xg for 10 min at 4 °C were used for Western blot analysis and duplex enzyme-linked immunosorbent assays. Protein concentration was measured using the Pierce bicinchoninic acid assay (Thermo Scientific, MA, USA).

### 2.4. Western blot analysis

Samples containing 40 *μ*g protein were fractionated by sodium dodecyl sulfate polyacrylamide gel electrophoresis (SDS-8% PAGE) under denaturing and reducing conditions along with prestained molecular weight standards. After electrophoretically transferring the proteins onto PVDF [55, 60], the membranes were blocked in Pierce Superblock (Thermo Scientific) for 30 min at room temperature with gentle agitation, and probed overnight (4 °C) with ASPH rabbit polyclonal antibody (1:1000) or A85G6 mouse monoclonal ASPH (5.80 *μ*g/mL) in Tris-buffered saline containing 0.05% Tween-20 and 0.5% bovine serum albumin (BSA) (TBS-T-BSA) [55]. After several washes in TBS-T, the membranes were incubated with HRP-conjugated anti-rabbit or anti-mouse antibody in TBS-T plus casein (1:10,000) for 1 h at room temperature. After thorough washing of the membranes (TBS-T with agitation), immunoreactivity was detected using Pierce Supersignal West Pico (Thermo Scientific) and film autoradiography. The membranes were then stripped and reprobed with antibodies to the P85 subunit of phosphoinositol-3-kinase (p85-PI3K) as a loading control and immunoreactivity was detected as described above. Signal intensities were quantified using ImageJ software.

### 2.5. Duplex enzyme-linked immunosorbent assays (ELISAs)

Direct binding duplex ELISAs were used to measure immunoreactivity to target proteins detected with horseradish peroxidase-conjugated secondary antibody and Amplex UltraRed soluble fluorophore (Invitrogen, Carlsbad, CA, USA) as described [35]. To adjust for variability in sample loading and binding to the wells, these results were normalized to large acidic ribosomal protein (RPLPO) (Proteintech Group Inc., Chicago, IL, USA) which was biotinylated and detected with streptavidin-conjugated alkaline phosphatase and the 4-methylumbelliferyl phosphate (4-MUP) substrate. Fluorescence intensities (Amplex Red: Ex 565 nm/Em 595 nm; 4-MUP: Ex360/Em450) were measured in a SpectraMax M5 (Molecular Devices, Sunnyvale, CA, USA). Antibody omission controls were included. The calculated target protein/RPLPO ratios were used for intergroup comparisons.

### 2.6. Statistics

Results were graphed using bar plots in which the means are depicted as horizontal bars, 95% confidence interval limits correspond to the upper and lower boundaries of the boxes, and the range is represented by the upper and lower stems. Intergroup comparisons were made using one-way or two-way analysis of variance (ANOVA) with Tukey or linear trend post hoc tests (GraphPad Prism 6, San Diego, CA, USA). *F*-ratios and *P*-values are tabulated. Significant (*P < .*05) and trend-wise (.05 *< P < .*10) post hoc test differences are shown in the graphs.

### 2.7. Materials

Pharmaceutical grade ethanol was used in the in vivo experiments. The A85G6 and A85E6 monoclonal antibodies to ASPH were generated to human recombinant protein [46] and purified over Protein G columns (Healthcare, Piscataway, NJ, USA). Otherwise, antibodies used for duplex ELISAs were purchased from Abcam (Cambridge, MA, USA). RPLPO antibody was from the Proteintech Group Inc. (Chicago, IL, USA). ELISA MaxiSorp 96-well plates were purchased from Nunc (Rochester, NY, USA). Horseradish peroxidase (HRP)-conjugated secondary antibody and Amplex Red soluble fluorophore were purchased from Invitrogen (Carlsbad, CA, USA). The SpectraMax M5 microplate reader was purchased from Molecular Devices Corp. (Sunnyvale, CA, USA). BCA reagents were from Pierce Chemical Corp. (Rockford, IL, USA). All other fine chemicals were purchased from CalBiochem (Carlsbad, CA, USA), Pierce (Rockford, IL, USA) or Sigma (St. Louis, MO, USA).

## 3. Results

### 3.1. Time-dependent effects of ethanol on the frontal cortex

**Figure 1:**
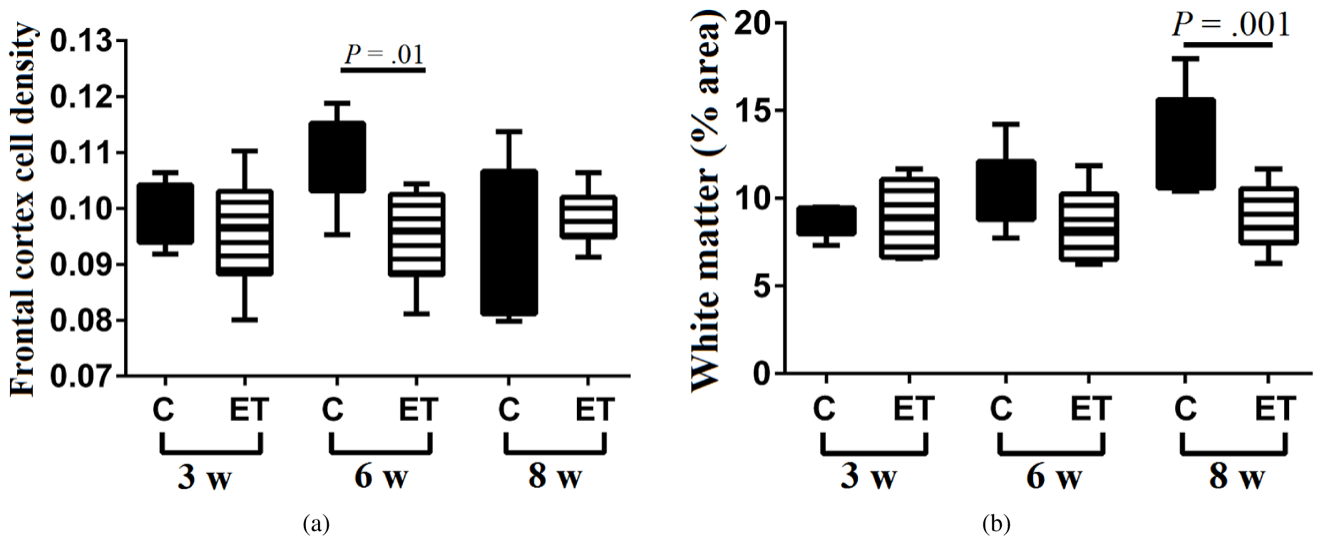
Ethanol inhibits frontal lobe white matter growth. Long Evans rats (6–8/group) were fed for 3, 6 or 8 weeks with isocaloric liquid diets containing 0% or 26% ethanol by caloric content, and two weeks prior to each endpoint, they were IP binged with 2 g/kg ethanol (ethanol group) or saline (control group) 3*×*/week (*N* = 6–8 rats/group). Formalin-fixed, paraffin embedded histological sections of frontal lobe were stained with Luxol fast blue, hematoxylin, and eosin (LHE) and used for image analysis of (a) cortical cell density and (b) relative (% of total) cross-sectional area of white matter. ANOVA with post hoc Tukey tests identified specific intergroup differences.

Standardized histological sections of frontal lobe and cerebellar vermis were used for image analysis to assess progressive changes in WM abundance following chronic+binge ethanol exposures. In the frontal cortex, cell density was measured in sections stained with Cresyl violet (Figure 1(a)) and percentage area occupied by WM was measured in adjacent sections stained with Luxol fast blue, hematoxylin, and eosin (LHE) (Figure 1(b)). Luxol fast blue selectively stains WM myelin blue. In control frontal lobes, the mean cortical cell density increased between Week 3 and Week 6, but declined to the Week 3 levels at the 8-week time point. In contrast, in the ethanol group, mean cortical cell density was unchanged over the course of the experiment. Intergroup statistical comparisons demonstrated that at the 3-week and 8-week time points, there were no significant effects of ethanol on neuronal density, but at the 6-week time point, cortical cell densities were significantly higher in the control brains (*P* = .01). The time-dependent modulation of cortical cell density in control brains could be attributed to increased populations of glial cells at Week 6, and expansion of the neuropil with growth of synaptic terminals between Weeks 6 and 8. The muted responses in ethanol-exposed brains could have been due to reduced dendritic arborization and synaptic growth/plasticity, which are recognized as neurotoxic/degenerative effects of alcohol [61,62,63].

### 3.2. Time-dependent effects of ethanol on frontal WM

The mean percentage cross-sectional area occupied by WM in the frontal lobe progressively increased over time in the control group but remained static in the ethanol-exposed group (Figure 1(b)). One-way ANOVA with post hoc linear trend analysis of the time-dependent increases in relative WM cross-sectional area revealed a Slope = 2.34 and an *R*^2^ = 0.461 (*P* = .0026) for the control group. In contrast, the corresponding Slope = −0.084 and *R*^2^ = 0.001 calculated for the ethanol group were not statistically significant. The mean relative cross-sectional area of frontal WM was significantly greater in the control versus ethanol-exposed group at the 8-week time point (*P* = .001).

### 3.3. Time-dependent effects of ethanol on the cerebellar cortex

**Figure 2:**
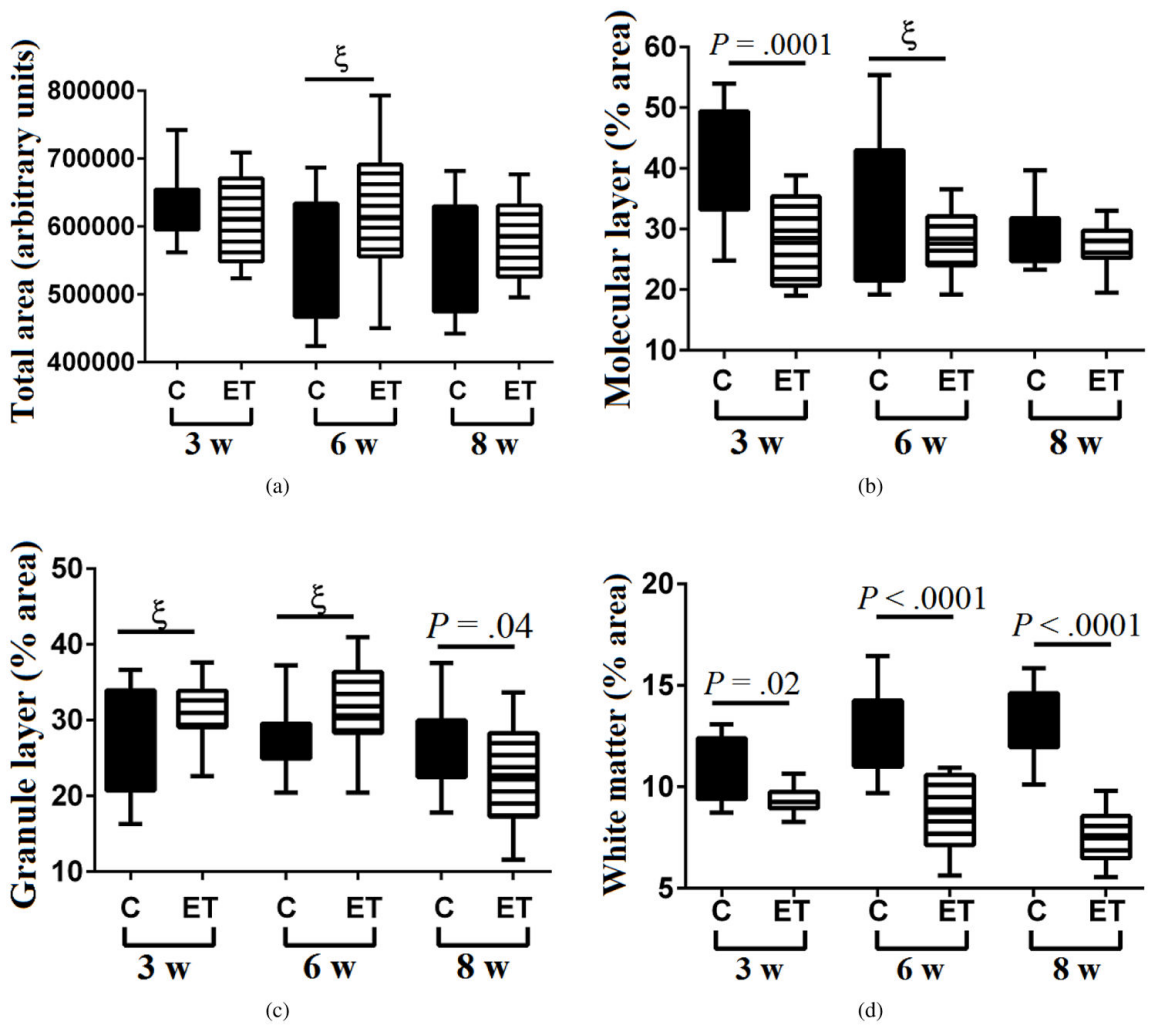
Effects of ethanol on cortical and white matter structures in the cerebellar vermis. Long Evans rats (6–8/group) were fed for 3, 6 or 8 weeks with isocaloric liquid diets containing 0% or 26% ethanol by caloric content, and two weeks prior to each endpoint, they were IP binged with 2 g/kg ethanol (ethanol group) or saline (control group) 3*×*/week (*N* = 6–8 rats/group). Formalin-fixed, paraffin embedded histological sections of cerebellar vermis were stained with LHE and used for image analysis (Image J) to determine the (a) overall cross-sectional area, (b) relative (% of total area) cross-sectional area of the molecular layer, (c) relative cross-sectional area of the granule cell layer, and (d) relative cross-sectional area of white matter. Bar plots depict means (horizontal bars), 95% confidence interval limits (upper and lower boundaries of the boxes), and range (upper and lower stems). ANOVA with post hoc Tukey tests identified specific intergroup differences. *ξ*= .05 *< P < .*10 (statistical trend).

The mean total cross-sectional area of the cerebellar vermis remained relatively unchanged in both control and ethanol groups over the time course of the study (Figure 2(a)). In control cerebella, the mean percentage area occupied by the molecular layer declined over time and was significantly greater than in the ethanol group at the 3-week (*P* = .0001) but not the 6- or 8-week time points. In contrast, ethanol exposure did not detectably modulate the mean percentage area occupied by the molecular layer over the course of the experiment (Figure 2(b)). Regarding the granule cell layer, the mean percentage areas were greater in the ethanol group at the 3- and 6-week time points. However, at the 8-week time point, the trends were switched such that the mean percentage area of granule cells was significantly lower in the ethanol-exposed relative to control group (*P* = .04) (Figure 2(c)).

### 3.4. Time-dependent effects of ethanol on cerebellar WM

In control cerebella, WM cross-sectional area expanded over time such that its mean percentage area increased with age (Slope = 1.18; *R*^2^ = 0.238; *P* = .0027). In contrast, ethanol exposures produced opposite effects, resulting in progressive reductions in the mean percentage area of WM over time (Slope = −0.91; *R*^2^ = 0.24; *P* = .0028) (Figure 2(d)). Correspondingly, the mean relative area of cerebellar WM was significantly and strikingly reduced relative to control at each of the three time points. The stepwise widening of the intergroup differences indicates that ethanol caused progressive cerebellar WM atrophy with increasing duration of exposure. Conceivably, the modest elevations in the mean relative area of the granule cell layer vis-à-vis fixed areas of the vermis could be attributed to corresponding WM atrophy in the ethanol group.

### 3.5. Ethanol inhibition of ASPH expression in WM

**Figure 3:**
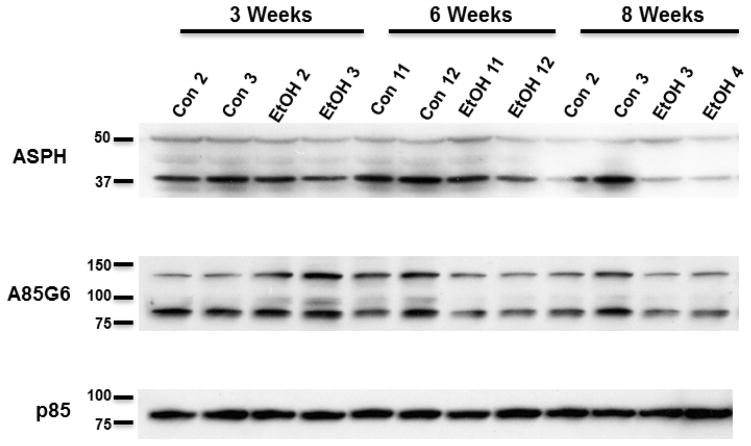
Effects of different durations of ethanol exposure on ASPH expression. Long Evans male rats (6–8/group) were fed for 3, 6 or 8 weeks with isocaloric liquid diets containing 0% or 26% ethanol, and two weeks prior to each endpoint, rats were IP binged with 2 g/kg ethanol (ethanol group) or saline (control group) 3*×*/week. Western blot analysis of frontal lobe white matter (representative samples shown) was performed with polyclonal anti-ASPH, and the monoclonal A85G6 antibodies to examine effects of different durations of ethanol exposure on ASPH protein expression. Polyclonal anti-ASPH antibody binds to the N-terminus of ASPH. However, that antibody also detect Humbug since its amino acid sequences are virtually identical to those in the N-terminal region of ASPH. A85G6 binds to the C-terminus of ASPH which contains a catalytic domain that is not present in Humbug. Both antibodies can detect cleavage products of ASPH and Humbug. Each lane corresponds to a different frontal lobe sample. After probing for ASPH, the blots were stripped and reprobed with antibodies to the p85 subunit of PI3 kinase as a negative control.

**Figure 4:**
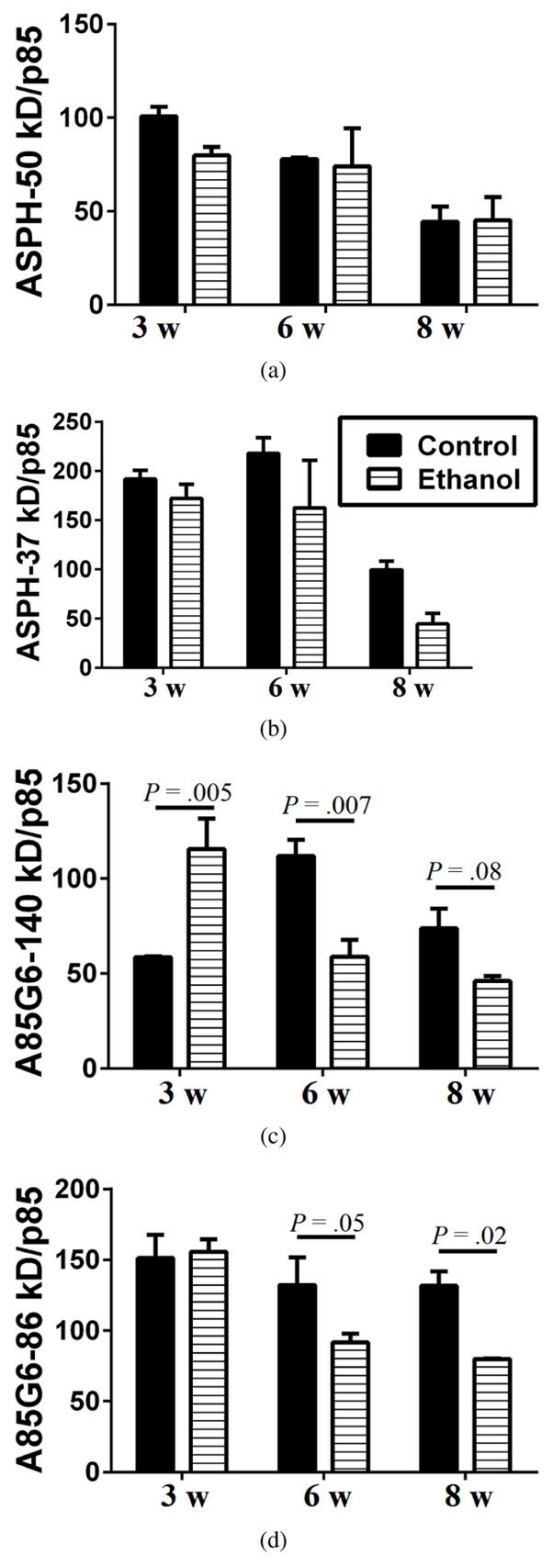
Digital quantification of Western blot signals. The average intensities of the ASPH-50 kD, ASPH-37 kD, A85G6-140 kD, A85G6-86 kD, and p85-PI3K bands were measured using Image J. Relative abundance of each protein was assessed by calculating the ratios of the ASPH/p85 and A85G6/p85 signals. Graphs depict mean *±*SEM of all samples analyzed in each group. Results were analyzed by two-way ANOVA tests with post hoc Tukey tests. Significant differences and statistical trends are indicated over the bars.

Western blot analysis was used to quantify ASPH expression in microdissected frontal WM using rabbit polyclonal ASPH and mouse monoclonal A85G6-ASPH antibodies. The polyclonal antibody mainly recognized *~* 50 kD and *~* 37 kD cleavage products of either ASPH or Humbug [55,64,65]. Humbug is a truncated protein corresponding to the N-terminal region of ASPH [49,50,51,66,67]. Its function is mainly related to calcium flux in the ER [67] and cell adhesion [36,52]. The *~* 50 kD species was similarly expressed in both groups across all time points, but expression significantly declined over time (*P* = .008) such that the mean levels were 40%–50% lower at the 8-week versus the 3-week time point (Figures 3 and 4(a)). The *~* 37 kD ASPH species was also more abundantly expressed at the 3-week and 6-week compared with the 8-week time point in both groups. However, the *~* 37 kD ASPH protein was more abundantly expressed than the *~* 50 kD species, and its decline in levels from the 3- and 6-week to the 8-week time point was statistically significant (*P* = .002) (Figures 3 and 4(b)). In addition, the mean relative expression of the *~* 37 kD ASPH was strikingly lower in the ethanol versus control group at the 8-week time point, although the difference was not statistically significant (Figures 3 and 4(b)).

With the A85G6-ASPH monoclonal antibody, which specifically binds to the C-terminal regions of ASPH and detects ASPH but not Humbug 46, both the *~* 140 kD phosphorylated form of ASPH and the *~* 86 kD native protein were detected (Figure 3). For the *~* 140 kD A85G6-ASPH, two-way ANOVA tests demonstrated significant exposure *×* time interactive effects (*P* = .01) and a trend effect for duration of ethanol exposure (*P* = .08). At the 3-week time point, the *~* 140 kD A85G6-ASPH was expressed at higher levels in the ethanol group (*P* = .005), but at the 6-week and 8-week time points, the expression levels were significantly (*P* = .007 at 6 weeks) or trend-wise (*P* = .08 at 8 weeks) reduced in the ethanol group (Figures 3 and 4(c)). For the *~* 86 kD A85G6-ASPH, two-way ANOVA tests demonstrated significant duration (*P* = .05) and exposure (*P* = .02) effects of ethanol. The highest levels of the *~* 86 kD A85G6-ASPH protein were measured at the 3-week time point in both groups (Figures 3 and 4(d)). In the control group, modest reductions in the mean level of the *~* 86 kD A85G6-ASPH occurred over time, whereas in the ethanol group, the expression levels were significantly reduced and lower than control at the 6- (*P* = .05) and 8-week (*P* = .02) time points. In essence, A85G6-ASPH expression in frontal lobe WM was significantly inhibited by ethanol after 6 or 8 weeks of exposure. In contrast, significant inhibitory effects of ethanol on ASPH proteins detected with the polyclonal antibody (which included Humbug+ASPH) were less striking and just marginally evident at the 8-week time point.

### 3.6. Ethanol effects on ASPH, Notch, Jagged, HES-1, and HIF-1*α* expression in frontal lobe white matter

**Figure 5:**
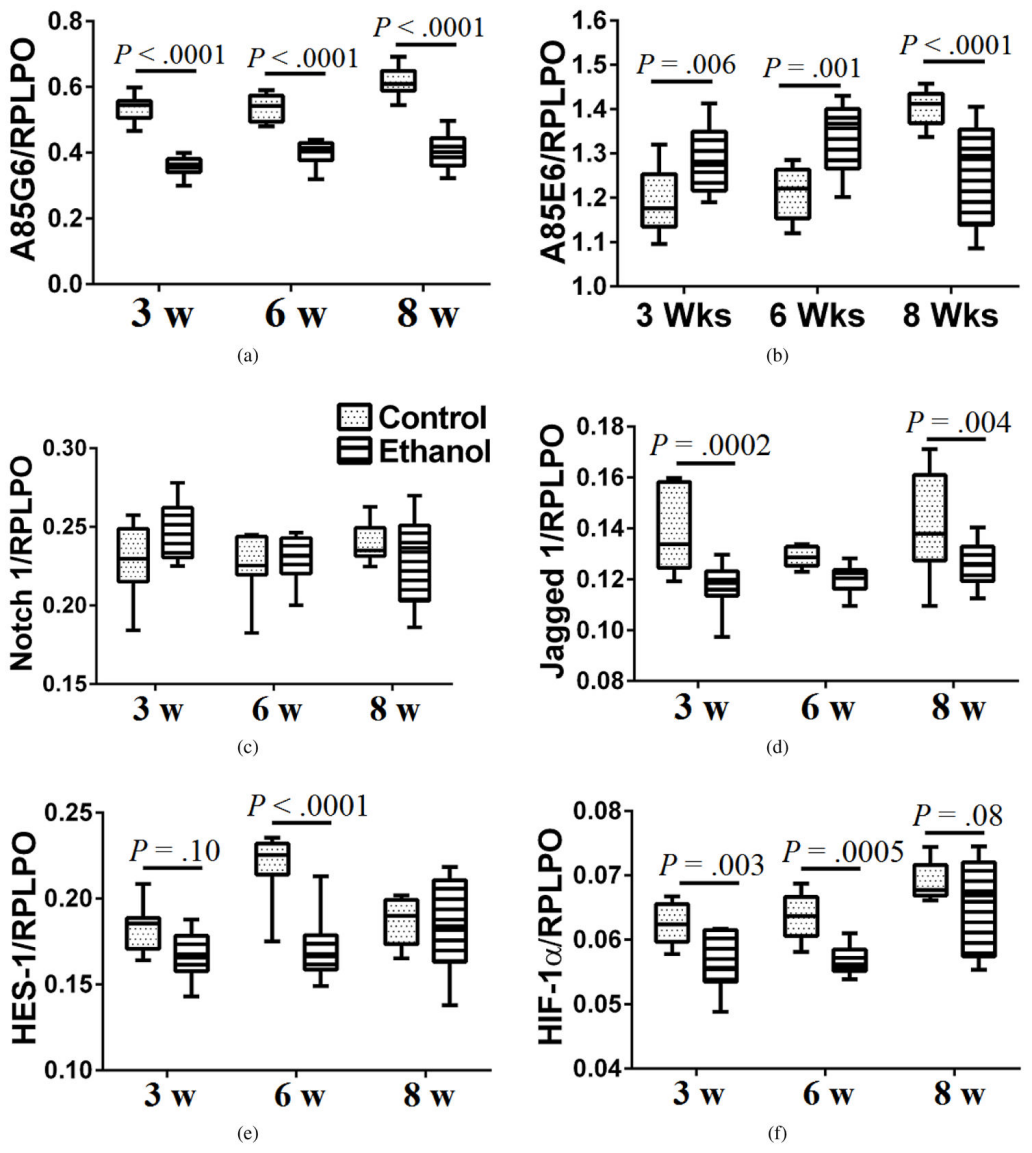
Duplex ELISAs were used to measure (a) ASPH using the A85G5 monoclonal antibody, (b) Humbug+ASPH, using the A85E6 monoclonal antibody, (c) Notch 1, (d) Jagged 1, (e) HES-1, and (f) HIF-1*α* (*N* = 6–8 samples/group). Immunoreactivity was normalized to RPLPO. Results were analyzed by two-way ANOVA (Table 1) and the post hoc Tukey test. Significant differences and statistical trends are displayed within the panels.

Duplex ELISAs were used to measure frontal WM immunoreactivity to A85G6-ASPH (Figure 5(a)), A85E6-ASPH (Humbug) (Figure 5(b)), Notch 1 (Figure 5(c)), Jagged 1 (Figure 5(d)), hairy and enhancer of split-1 (HES-1; Figure 5(e)), and HIF-1*α* (Figure 5(f)), with results normalized to RPLPO. Corresponding with the Western blot results, A85G6-ASPH expression was significantly reduced by ethanol at each time point. In contrast, A85E6-ASPH (Humbug) expression was significantly elevated in the ethanol group at the 3- and 6-week time points, but significantly reduced at the 8-week time point. These findings correspond with the Western blot results obtained with the polyclonal ASPH antibody (see Figure 3). Notch 1 expression was similar across all time points and was not significantly modulated by ethanol exposure (Figure 5(c)). In contrast, the mean levels of Jagged 1 expression were consistently lower in ethanol-exposed samples. The intergroup differences were statistically significant at the 3-week and 8-week time points (Figure 5(d)). HES-1 expression was modulated with time (age) and ethanol exposure. At the 3-week time point, ethanol caused trend reductions in mean HES-1 expression, but at the 6-week time point, the intergroup difference was highly statistically significant, mainly due to increased levels in the control group. At the 8-week time point, HES-1 expression was similar in control and ethanol-exposed frontal WM tissue. HIF-1*α* expression was also inhibited by ethanol. The intergroup differences were statistically significant at the 3-week and 6-week time points, but just reached a statistical trend at the 8-week time point (Figure 5(f)). Therefore, ethanol inhibition of ASPH expression was associated with reduced expression of Notch pathway proteins (i.e., Jagged 1 and HES-1, and HIF-1*α*) which crosstalk through Notch signaling [54,57].

## 4. Discussion

This study examined the time-dependent effects of chronic+ binge ethanol exposures on the frontal lobe and cerebellar vermis of adult Long Evans male rats. The model utilized high levels of chronic ethanol feeding with superimposed binge administrations to simulate the human condition associated with alcoholic liver and brain diseases. This model produces significant alcohol-related liver injury with steatohepatitis [68,69] as well as deficits in spatial learning and memory [44,70]. The main objective in these studies was to characterize progressive ethanol effects in two brain regions that are reproducibly damaged in adult humans and experimental animals with alcohol-related neurodegeneration. The emphasis was on WM because although WM atrophy and degeneration are well-recognized features of alcoholic brain disease, their onset and rate of development have not been fully evaluated.

In control frontal cortex, the time-dependent increases in cell density between Weeks 3 and 6 were likely due to increased glia, whereas the relative reductions between Weeks 6 and 8 could be explained by expansion of the neuropil associated with ongoing dendritic spine growth [71]. The absent net response in ethanol-exposed brains could reflect combined effects of neuronal loss with expansion of glia between Weeks 3 and 6, and muted dendritic arborization between Weeks 6 and 8.

Image analysis of the cerebellar vermis demonstrated no significant intergroup differences in the overall cross-sectional areas. Instead, the main differences were attributable to early (Weeks 3 and 6) relative reductions in the molecular layer and late reductions in the granule cell layer in ethanol-exposed cerebella. The molecular layer contains predominantly nerve terminals from the granule cell layer. Therefore, the early reductions in relative area of the molecular layer suggest that ethanol exposure causes loss of nerve terminals destined to synapse on Purkinje cells which drive motor output from the cerebellum. These adverse effects could account for alcohol-related cerebellar dysfunction, manifested by poor performance on tasks such as the rotarod [40,72,73]. The relative decline in granule cells late in the time course (Week 8), corresponds with granule cell loss that is characteristic of alcohol-related cerebellar degeneration in humans and experimental models [8,37,74]. These findings suggest that cerebellar degeneration may be partly reversible prior to granule cell loss (i.e., the 8-week time point) since their preservation vis-à-vis abstinence would enable potential recovery and neurite regeneration.

Image analysis demonstrated significant progressive relative increases in the cross-sectional area of control WM but no changes over time in the ethanol group. These data indicate progressive expansion of frontal lobe WM volume with increasing age in controls, and no net growth in ethanol-exposed brains. The inhibitory effects of ethanol on cerebellar WM were more striking than in the frontal lobe. While control cerebellar WM progressively expanded over time, it significantly declined in the ethanol-exposed group. Therefore, ethanol not only inhibited WM growth, it also caused atrophy in the cerebellum.

Cerebellar WM contains both afferent and efferent myelinated fibers. Their progressive degeneration from the earliest time points suggests that motor relay functions are substantially impaired even after a relatively short period of heavy alcohol abuse. Since WM is largely composed of myelin, impaired function of oligodendrocytes could account for deficits in myelin biosynthesis and maintenance or disproportionately increased myelin degradation vis-à-vis normal rates of biosynthesis. The greater severity of ethanol-induced damage to cerebellar versus frontal lobe WM suggests that oligodendrocyte susceptibility to injury and degeneration also vary with brain region. The findings herein indicate differential regional adverse effects of ethanol on WM integrity and highlight the concept that target vulnerability varies. Correspondingly, regional differences in oligodendrocyte vulnerability to injury and associated demyelination have been described in an experimental cuprizone exposure model [75].

Previous studies demonstrated that ethanol impairs insulin and IGF-1 signaling in neurons and gray matter structures in the brain, and that these responses are associated with reduced expression and function of insulin/IGF-1 target genes and proteins, including ASPH [36,46]. In addition, crosstalk between ASPH and Notch networks is disrupted following chronic experimental ethanol exposures [57]. Studies of ethanol’s effects on ASPH and Notch networks were extended to the present model to gain a better understanding of the molecular mechanisms of alcohol-induced progressive WM atrophy. The experimental approaches were driven by prior evidence that (1) insulin/IGF signaling regulates oligodendrocyte survival and function [27,28,29,30], (2) WM degeneration in human CADASIL has been linked to reduced insulin/IGF and Notch pathway signaling [58], (3) experimental chronic ethanol exposures lead to substantial impairments in oligodendrocyte function, myelin integrity, and myelin maintenance associated with inhibition of insulin/IGF signaling through survival and metabolic pathways [8,21, 70,76], and (4) ASPH functions via crosstalk with Notch networks including HIF-1*α* [54,57].

Western blot analyses of frontal WM demonstrated significant inhibitory effects of ethanol on ASPH expression, with larger effects at Week 8 compared with Week 6, corresponding with the progressive relative reductions in WM area. However, at the 3-week time point, the minimal alterations in ASPH expression correspond with the absence of detectable ethanol effects on WM structure. ELISA studies utilized the A85G6 and A85E6 monoclonal antibodies which respectively recognize ASPH-86 kD and ASPH+Humbug [46]. However, since Humbug is 7- to 10-fold more abundant than ASPH, combined detection of ASPH with Humbug can produce mixed net responses. Importantly, ASPH expression, specifically detected with the A85G6 antibody, was significantly inhibited at all time points. In contrast, ASPH+Humbug immunoreactivity was significantly elevated in the earlier time points but suppressed by Week 8. These findings indicate that ethanol has profound inhibitory effects on WM ASPH expression, even after relatively short-term exposures, but its inhibitory effects on WM Humbug occur after long-term exposures.

ASPH’s function has mainly been linked to cell motility in immature neurons and a broad range of malignant neoplastic cell types [49,51,52,53,77,78,79,80,81,82]. Humbug has roles in regulating calcium flux and cell adhesion [52,67]. Although their functions in oligodendrocytes and myelin have not yet been determined, potential clues stem from independent studies showing that inhibition of ASPH causes cellular senescence [56]. Therefore, ethanol inhibition of ASPH may mediate WM atrophy and degeneration by causing senescence of oligodendrocytes, impairing their capacity to maintain myelin.

Previous studies linked ASPH’s function through Notch and Jagged [52,54,57] which have consensus sequences for ASPH hydroxylation [51]. To demonstrate how ethanol inhibition of ASPH expression impacts Notch networks in WM, we measured Notch 1, Jagged 1, HES-1, and HIF-1*α* immunoreactivity. All except for Notch were inhibited by ethanol with intergroup differences achieving statistical significance or a statistical trend at most time points. Although Notch protein was not reduced, its function could still have been impaired due to decreased hydroxylation, cleavage, and translocation to the nucleus. Notch signaling plays key roles in gliogenesis and glial differentiation [83,84], and impairments in Notch inhibit nerve regeneration [85]. Inhibition of the HES-1 transcription factor leads to reduced expression of downstream target genes including regulators of the cell cycle [86]. In addition, suppressing HES disrupts neurodevelopment and histogenesis, and accelerates cell differentiation leading to increased gliogenesis [86].

HIF-1*α* inhibition was most pronounced after 3 and 6 weeks of ethanol exposure, paralleling the HES-1 responses. Previous studies linked Notch activation to HIF-1*α* expression [54] and hypoxia [87]. Increased levels of HIF-1*α* have been associated with hypoxia-type myelin and oligodendrocyte loss in the brain [88]. Of note is that increased activation of PI3K/Akt is associated with increased expression of HIF-1*α* [89], but following ethanol exposure, PI3K/Akt signaling is inhibited [37,40,45], corresponding with the reduced HIF-1*α* expression measured in frontal lobe WM. In addition, there is some evidence that HIF-1*α* regulates expression of oligodendrocyte lineage gene-1 in mediating myelin repair following injury [90]. More recently, HIF-1*α* expression has been linked to oligodendrocyte precursor cell maturation and WM myelination, and coupling of these functions with axonal integrity and angiogenesis in the forebrain [91]. Therefore, the reduced HIF-1*α* expression measured in ethanol-exposed WM most likely contributed to the deficiencies in myelin and axonal maintenance that led to WM atrophy and degeneration.

The findings in these experimental/preclinical studies provide new clues about the mechanisms of alcohol-related WM degeneration. Importantly, they demonstrate that WM atrophy is associated with reduced expression of ASPH and Humbug, which are downstream targets of insulin/IGF-1 stimulation. Moreover, these studies link alcohol-related WM degeneration to impairments in Notch and HIF-1*α* signaling networks. Since previous studies reported very similar abnormalities in neurons and cortical structures following chronic or chronic+binge ethanol exposures [36, 46,57], it is likely that impairments in brain insulin/IGF-1 signaling through ASPH, Notch, and HIF-1*α* pathways are at the core and mechanistically link the molecular pathogenesis of neuronal and oligodendroglial cell degeneration. In the cortex, the consequences of these impairments include reduced neuronal plasticity, whereas in WM, the adverse effects lead to loss of myelin homeostasis, increased myelin lipid breakdown, lipid peroxidation, oxidative stress, and ultimately impaired neuronal conductivity with declines in executive function. However, from the standpoint of therapeutics, a parsimonious approach that globally supports insulin/IGF-1 pathways in the human brain could potentially provide optimum neuroprotection for both neurons and oligodendrocytes in the context of alcohol use disorders.

## Figures and Tables

**Table 1:** Ethanol *×* duration effects on ASPH and related signaling molecules (two-way ANOVA).

Protein	Ethanol factor		Time factor		Ethanol *×* time	

	*F*-ratio	*P*-value	*F*-ratio	*P*-value	*F*-ratio	*P*-value
	
A85G6-ASPH	30.45	*< .*0001	207.7	*< .*0001	3.10	.058
A85E6-ASPH	1.68	N.S.	13.78	*< .*0001	34.82	*< .*0001
Notch 1	1.28	N.S.	0.88	N.S.	2.43	.10
Jagged 1	66.79	*< .*0001	3.11	.05	1.42	N.S.
HES-1	16.30	.0006	9.24	.0004	11.35	.0001
HIF-1*α*	27.54	*< .*0001	25.41	*< .*0001	0.823	N.S.

Frontal lobe white matter homogenates were used in duplex ELISAs with immunoreactivity normalized to RPLPO (see Section 2). Data were analyzed by two-way ANOVA with the Tukey multiple comparison post hoc test. Corresponding data are graphed in Figure 5.
